# Cavernous sinus thrombosis in ulcerative colitis recurrent after pregnancy: A case report

**DOI:** 10.1002/ccr3.7081

**Published:** 2023-03-08

**Authors:** Sepideh Akhoundizardini, Reyhaneh Jafarshad, Azadeh Taftian, Matineh Nirouei, Hamid Mahboubipour, Masoumeh Farahani

**Affiliations:** ^1^ Alborz University of Medical Sciences Karaj Iran; ^2^ Gastroenterology Department Imam Khomeini Complex Hospital, Tehran University of Medical Sciences Tehran Iran; ^3^ Gastroenterologist Alborz University of Medical Sciences Karaj Iran; ^4^ Gynecologist, Assistant Professor of Alborz University of Medical Sciences Department of Obstetrics and Gynecologists Alborz University of Medical Sciences Karaj Iran

**Keywords:** inflammatory bowel disease, pregnancy, thrombosis

## Abstract

Inflammatory bowel disease and pregnancy are risk factors for increased hyper coagulopathy state. A 35‐year‐old woman with ulcerative colitis was presented in this study. She had the recurrence of the disease during pregnancy. She suffered cavernous sinus thrombosis simultaneously.

## Graphical abstract

Increased coagulation occurs in pregnant or postpregnancy patients. Also, patients with IBD are at risk of coagulation more than others. This causes the prevalence of PTE and DVT to be higher in pregnant patients with IBD.

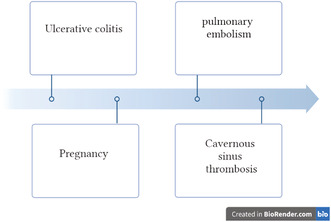

## 1 INTRODUCTION

Inflammatory bowel disease (IBD) is a risk factor for thrombotic complications, using multifactorial mechanisms such as platelet activation, increased fibrinogen, and coagulation factors 2, 5, 7, 8, 10, and 11 and destruction of endothelial function.^1^, ^2^

Pregnancy is a risk factor to increase coagulation in patients due to the change in procoagulation factors in the hemostatic system and the reduction in anti‐coagulation factors such as protein C, S; especially, the increased risk of coagulation in the pelvic vessels happens due to the rise in blood pressure.^3^ Therefore, pregnancy is a risk factor in developing venous thromboembolism (VTE), including DVT and PTE. Although pregnant patients with IBD are at risk of increased hyper coagulate state, it is a known leading cause of maternal mortality.^3^, ^4^, ^5^

The risk of increased coagulation in pregnant patients with IBD is high, but happening in the CNS vessels is rare and severe. Around 1% of patients suffer from thrombosis of the CNS vessels. Although the patients usually have symptoms such as seizures, headaches, variable levels of unconsciousness, and papillary edema, these patients are typically diagnosed late and have a poor prognosis.^6^, ^7^

## 2 PATIENT INFORMATION

**FIGURE 1:**
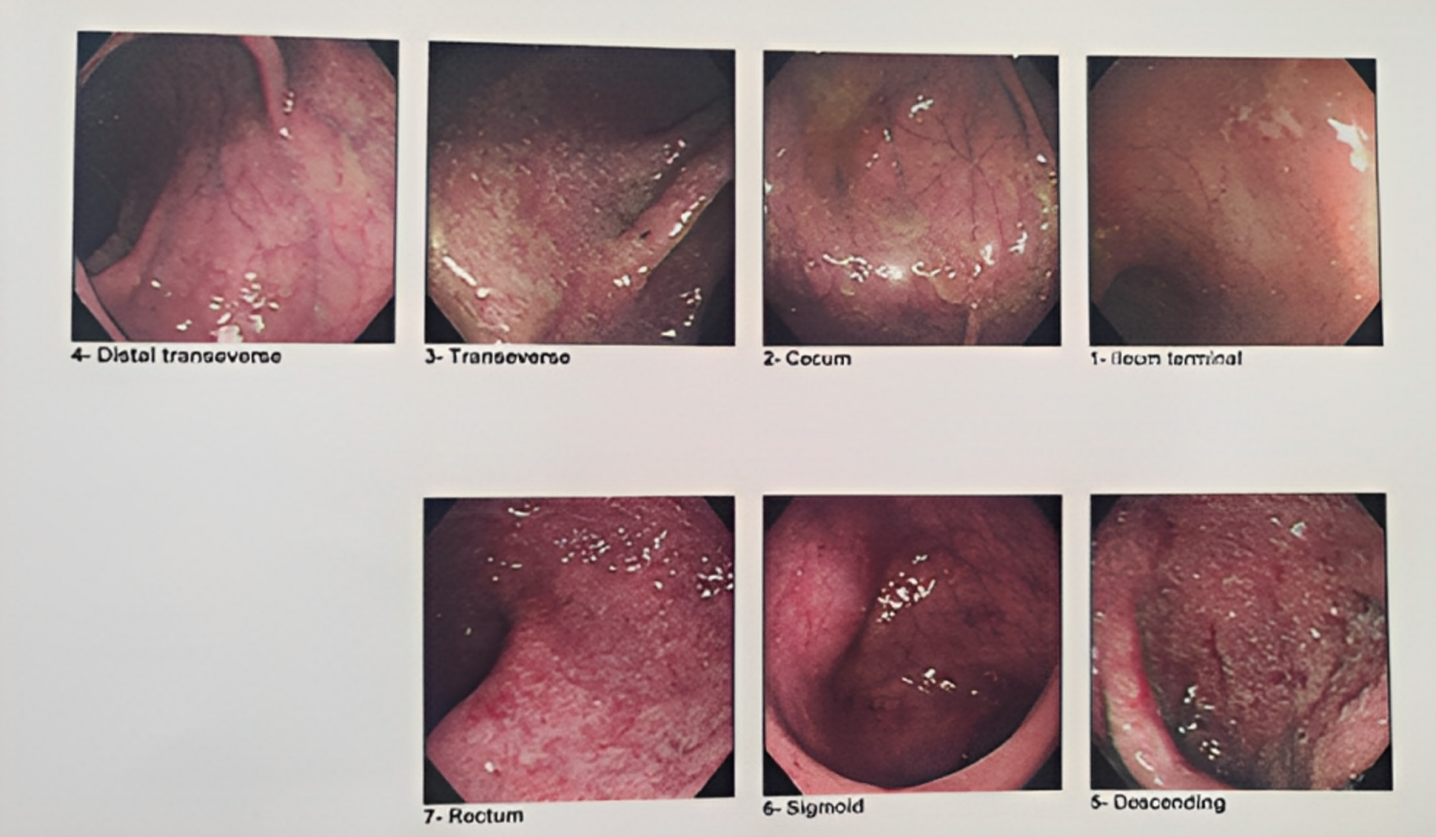
Endoscopy of the patient that demonstrated diffuse and fine mucosal ulcerative with partially decreased vascular pattern and without obvious mucosal friability (from the rectum up to the cecum). Multiple discrete ulcers and mucosal congestion were noted in the terminal ileum. These findings were compatible with diffuse ulceration colitis and backwash ileitis versus determinate colitis in Crohn's.

A 35‐year‐old Iranian woman, G3Ab2, presented due to intrauterine growth restriction (IUGR) and disappearance of the end‐diastolic wave of the umbilical artery in the sonography. She was at 32 weeks, and an emergency cesarean section was done. She had ulcerative colitis for 15 years. Her disease flared up during pregnancy. Figure 1 showed her colonoscopy which demonstrated an ulcerative colitis diagnosis. She has been treated with mesalazine, aspirin, and enoxaparin since the beginning of pregnancy.

Also, her sister has a history of ulcerative colitis, and her disease has been silent for a lifetime. However, her illness relapsed during her pregnancy.

## 3 CLINICAL FINDINGS

Two months after delivery, the patient presented with cough, shortness of breath, mild respiratory distress, headache, fever, myalgia, tachycardia, and right leg pain. The patient mentioned that her headache has gradually increased since childbirth. The patient's neurological examination was normal.

## 4 DIAGNOSIS ASSESSMENT

The patient's EKG (electrocardiography) was normal without any pathological findings. But in her laboratory tests, a positive D‐dimmer was reported. Her laboratory test revealed: platelet: 135000/mm3, PT: 13 s, PTT: 26 s, and INR: 1.1. The thrombophilia screening test was done, and the test result was negative. Arterial and venous color Doppler ultrasound of both lower limbs was performed, and no evidence of thrombosis or obstruction was observed. In the abdomen ultrasound, a cyst without septa and a solid component, size 27 × 28 mm, was observed in the right ovary.

**FIGURE 2:**
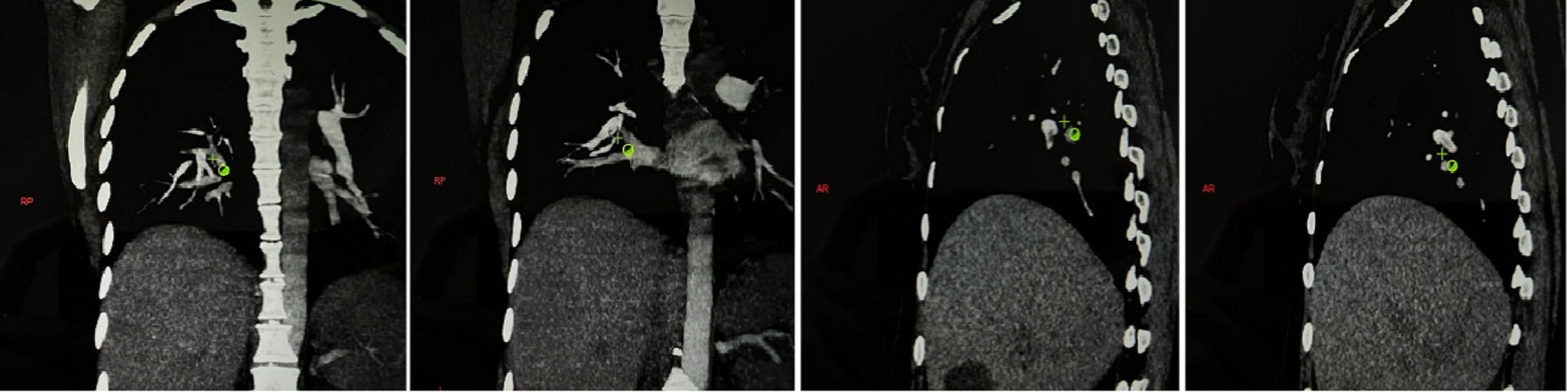
Partial thrombosis which started from the inferior lumbar artery with expansion to proximal medial branches and right posterior inferior basal segmental.

**FIGURE 3:**
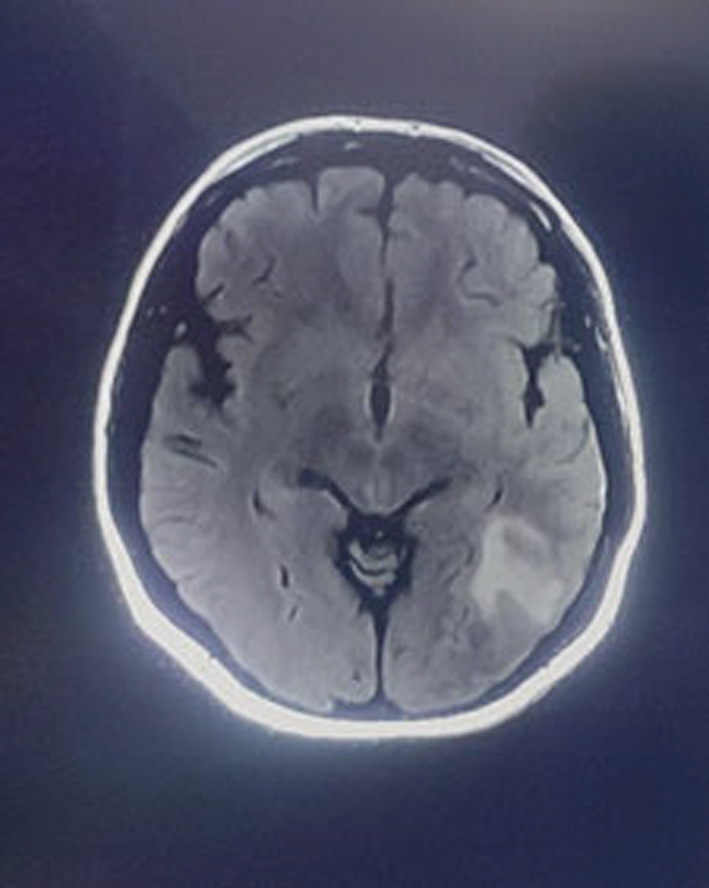
Axial brain CT scan showed hyperdensity in occipital lobe in favor of parenchymal bleeding.

**FIGURE 4:**
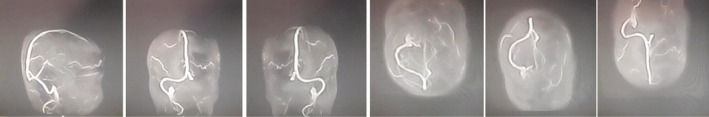
Lack of flow‐related enhancement was observed as suspicious for venous thrombosis.

**FIGURE 5:**
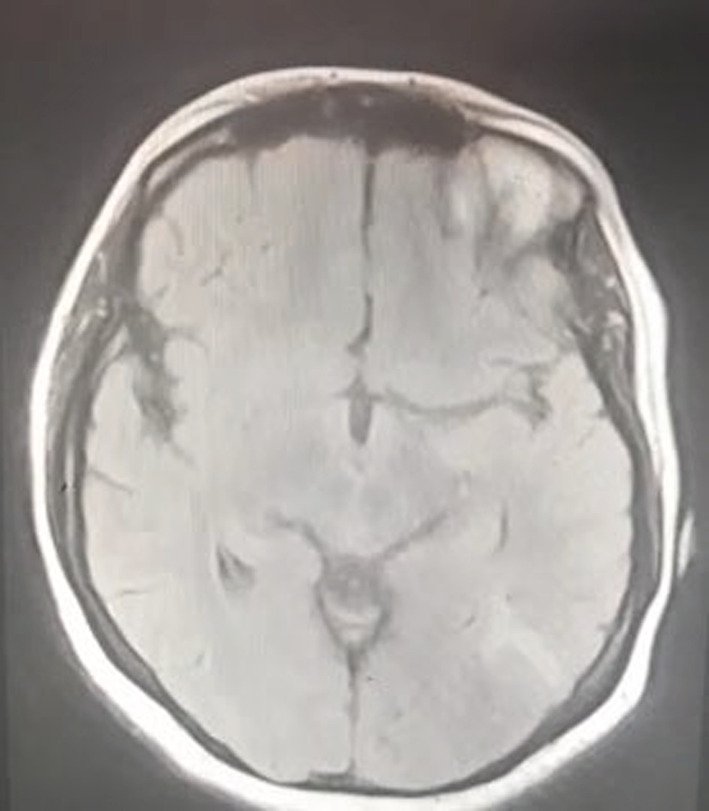
T1 signal hyperintensity which is suggestive of a brain hemorrhage.

Her chest X‐ray was normal. CT angiography reported a partial filling defect in the arteries of the inferior lobe in the right lung. The diagnosis of pulmonary embolism (PE) in the inferior lobe of the right lung was confirmed, and Figure 2 provided the CT angiography of the patient. CT scan of the patient's brain had hyperdensity in the left occipital lobe; Figure 3 showed her brain CT scan. Her brain MRV imaging revealed a lack of flow‐related enhancement, which was suspicious for venous thrombosis (Figure 4). Her brain MRI showed abnormal signal changes at the left temporal lobe related to intraparenchymal hemorrhage accompanied by mild edema (Figure 5).

## 5 THERAPEUTIC INTERVENTION

The patient was transferred to the intensive care unit (ICU). She was diagnosed with PTE and sinus thrombosis simultaneously. She was treated with anticoagulant drugs such as heparin and warfarin. The patient was treated with the anticonvulsant drug and was discharged after a week.

## 6 FOLLOW‐UP AND OUTCOMES

After 2 months, she went to the hospital with abdominal pain extending to the shoulder. The next day she had a laparotomy for a hemorrhagic cyst. During the surgery, the third space had 3.5 liters of blood. Because her INR was around 4, until 4 days after the surgery, heparin was replaced with warfarin. After 2 days, her INR reached approximately 2.5. Then, warfarin was started, and heparin was stopped. Finally, the patient was discharged in good general condition.

## 7 DISCUSSION

Despite the known relationship between inflammation and pregnancy with increased coagulation, most thromboses occur as VTE, although thrombosis in CNS vessels is rare,^7^ which has occurred in our patient.

Thrombosis of cerebral veins is a disease with a prevalence of 0.2 to 1.32 patients per 100,000 people. The majority of this disease in women, especially during pregnancy or after childbirth, is about twice compared with men.^8^

Risk factors such as contraceptive pills, IBD, cigarette smoking, and cancer could cause thrombosis.^9^ The relationship between thrombosis and colitis has not been conclusively proven, but new evidence suggests that ulcerative colitis is a risk factor for coagulation.^10^ The exact cause of this phenomenon is unknown, but some aspects, such as genetic factors and increasing coagulation factors due to inflammation, are suggested. When the disease is in a flared phase, there is an increased risk in the likelihood of coagulation.^11^ The patient presented in the present study was in the flared phase and suffered from sinus thrombosis and PTE after delivery.

The symptoms of sinus thrombosis in patients are very different; however, the most common symptom observed is headache, which was also observed in the present patient. Headache is usually limited to a specific area, but sometimes it is not.^9^ Motor weakness, mono paresis, and hemiparesis are observed in patients. Thirty‐nine percent of patients also experienced convulsion,^12^ which was not observed in the present patient.

A brain CT scan without contrast is the primary imaging that should be performed in patients with neurological symptoms. Around 30% of patients show abnormality in this imaging in the form of hyperdensity of cortical veins or dural venous sinuses. Also, a CT scan of our patient revealed hyperdensity in the occipital lobe.

Brain MRI and MRV, which are more sensitive than brain CT scan, should also be performed because they significantly affect early diagnosis. MRI and MRV are the best diagnostic methods for venous sinus thrombosis.^13^

If the disease was not diagnosed in time, the death rate was very high.^9^ Therefore, for our patient, MRI and MRV were performed immediately after the symptoms of respiratory distress and headache despite normal neurological symptoms.

## AUTHOR CONTRIBUTIONS

**Sepideh Akhoundizardini:** Conceptualization; data curation; investigation; project administration; writing – original draft. **Reyhaneh Jafarshad:** Conceptualization; data curation; methodology; project administration; supervision. **Azadeh Taftian:** Conceptualization; data curation; methodology; project administration. **Matineh Nirouei:** Conceptualization; investigation; methodology; project administration; writing – review and editing. **Hamid Mahboubipour:** Writing – review and editing. **Masoumeh Farahani:** Conceptualization; data curation; investigation; methodology; project administration; supervision; writing – review and editing.

## FUNDING INFORMATION

This work has no funding.

## CONFLICT OF INTEREST STATEMENT

The authors declare that they have no conflicts of interest.

## CONSENT

Written informed consent was obtained from the patient.

## Data Availability

Data sharing is not applicable to this article as no datasets were generated or analyzed during the current study.
